# A Thickness‐Invariant Origami Robot With Integrated Actuation and Multimodal Locomotion

**DOI:** 10.1002/advs.77418

**Published:** 2026-08-30

**Authors:** Davis Wing, Sam Bridge, William Bowler, Nathan Usevitch, Larry Howell

**Affiliations:** ^1^ Department of Mechanical Engineering Brigham Young University Provo Utah USA

**Keywords:** optimization, origami, robot, robotics, Yoshimura

## Abstract

Origami robots possess the potential for advanced reconfigurability in various contexts and environments. This paper describes and demonstrates the function of an origami robot following a thickness‐invariant version of the Yoshimura tessellation. This thickness‐invariance allows for the simple folding pattern to be expanded vertically, allowing all robotics components to be housed within the panels of the robot without impacting the functional relationships between folds of the pattern, or requiring other, more complex forms of thickness accommodation. The mathematics and engineering to create such a robot are presented to enable the use of these techniques in creating other origami‐based robots. This paper communicates methods for generating gait policies for origami‐based patterns, the limitations of such methods, and demonstrates such policies on a physical robot.

## Graphical abstract

A thickness‐invariant variation on the Yoshimura origami pattern is used as the basis of a robot, allowing power, actuation, and control systems to be embedded within the thickened panels of the pattern. The robot uses multiple kinematic branches to achieve distinct modes of locomotion, such as walking, running, galloping, and turning.

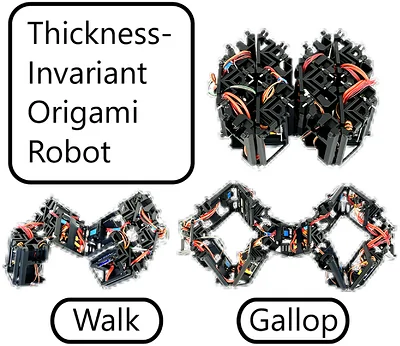

## 1 Introduction

Origami robots combine advantages in stowability, reconfigurability, and multifunctional motion. Based on the art of paper folding, classical origami assumes that the substrate material has negligible thickness, but robotic embodiment requires thickness for structure, actuation, and onboard electronics. Here, we show that those origami patterns, which display a property we call “thickness‐invariance” can serve as a promising robotics architecture. Using a folding pattern derived from a modified Yoshimura tessellation, we develop an untethered robot in which all core hardware is embedded within the folding body. This body can be of arbitrary thickness while still preserving identical geometric relationships between the fold angles from the zero‐thickness model. This means that beyond the zero‐thickness fold equations, no extra calculation or thickness accommodation is necessary to program pattern motion. Building a thick origami robot also introduces new challenges, such as how to coordinate the motions of the panels and how to determine which possible motions contribute to high‐level tasks like locomotion. We study how to coordinate the different actuators of the robot, as well as showing how the robot's kinematic branching gives rise to different locomotion modes. This work positions thickness‐invariant origami as a route to multifunctional, untethered robotic systems.

Origami‐inspired robots offer the ability to stow compactly, change shape to fit different use cases, and exploit multiple movement modes for different contexts or kinds of terrain. One key challenge facing researchers who wish to adapt origami patterns for robotics is thickness accommodation; many origami patterns struggle to fully fold or extend when made from thickened materials. While several techniques exist for accommodating thickness in various patterns, some patterns have been shown to possess the unique property of accommodating their own thickness, regardless of the patterns' dimensions. When mountain folds are applied to one side of the thickened structure and valley folds to the other, a few specific patterns, like the Yoshimura tessellation, show no changes or restrictions in hinge mobility. These “thickness‐invariant” patterns are particularly interesting for robotics applications, as they allow for energy storage media, actuators, and microprocessors to all be placed within the panels of the pattern itself, allowing for the design of untethered, self‐contained origami robots.

### 1.1 Concept Overview: Thickness‐Invariant Origami Robot

**FIGURE 1:**
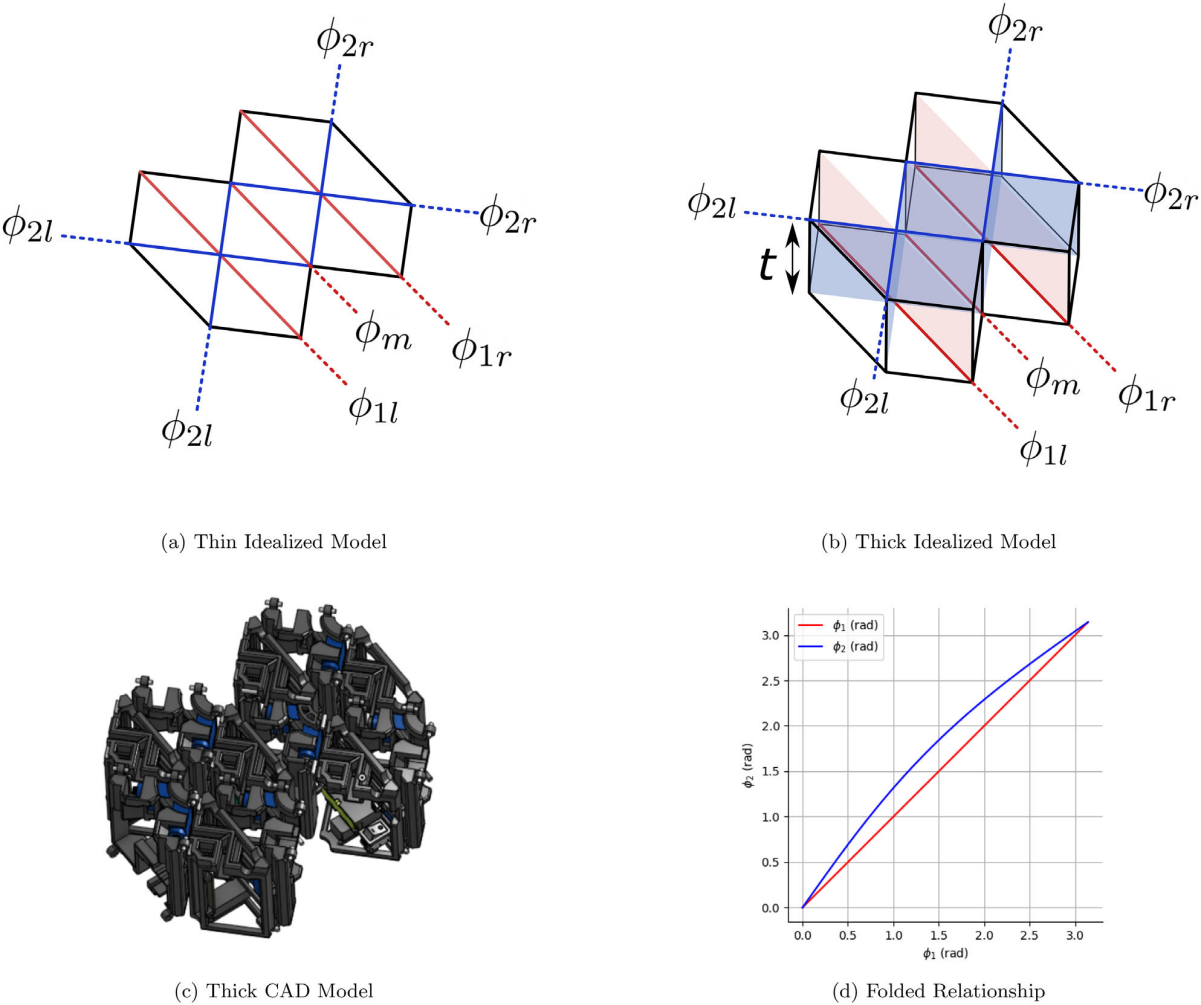
(a) A Yoshimiura folding pattern for a flat sheet, with dihedral angles labeled. The pattern is divided into a left half ($\phi_{1l}$, $\phi_{2l}$) and a right half ($\phi_{1r}$, $\phi_{2r}$), with a single hinge in the middle, $\phi_{m}$. (b) The modified Yoshimura pattern, thickened to the proportions used in this paper. All folds from the thin origami pattern are preserved in the thick pattern, but the mountain folds of the model lie on the bottom layer, and the valley folds lie on the top. (c) The CAD model of the robot, which achieves the same placement of hinges as the thick model. (d) The functional relationship between any $\phi_{1}$ (shown in red), and $\phi_{2}$ (shown in blue), per Equation (1).

We present a thick origami robot built from 12 rigid panels (see Figure 1). This robot is based on the Yoshimura pattern but is modified through the addition of arbitrary thickness. All folds from the flat pattern are preserved, but the mountain folds are moved to the bottom plane of the thickened structure, while the valley folds are moved to the top plane. This modified Yoshimura maintains the same dihedral angles as the zero‐thickness origami version but introduces cuts (kirigami) in the vertices between folds. Thus, as the pattern unfolds, the relationships between the dihedral angles remain identical to the flat origami, which we denote as “thickness‐invariance” [1]. This arbitrary thickness allows for power, actuation, and computation components to be placed within the panels of the pattern.

The properties of the thickness‐invariant Yoshimura pattern utilized by this paper can be generalized to other patterns. The properties implemented here are: constant thickness across all panels, the dihedral angles' invariance to pattern thickness, and the pattern's ability to tessellate. These factors allow for a construction consisting of similarly shaped panels of uniform thickness, which internally integrate all necessary robotics components and are modular in design. These are key features of this robot, and the principles can be extended to other designs to gain similar benefits. Interestingly, only the dihedral angles' invariance to pattern thickness is required for an origami robot like ours: constant thickness and pattern tessellation are elegant, but not strictly necessary. Thus, to construct an origami‐based robot with local, structural characteristics like ours, only the principles outlined in [1] must be observed (i.e., ensuring that all vertices of the thickened origami pattern embed a special case of spatial linkage), such as can  be found in [2, 3]. Similarly shaped panels and uniform thickness, as demonstrated here, provide additional benefits beyond what is minimally required to achieve the desired motion.

**FIGURE 2:**
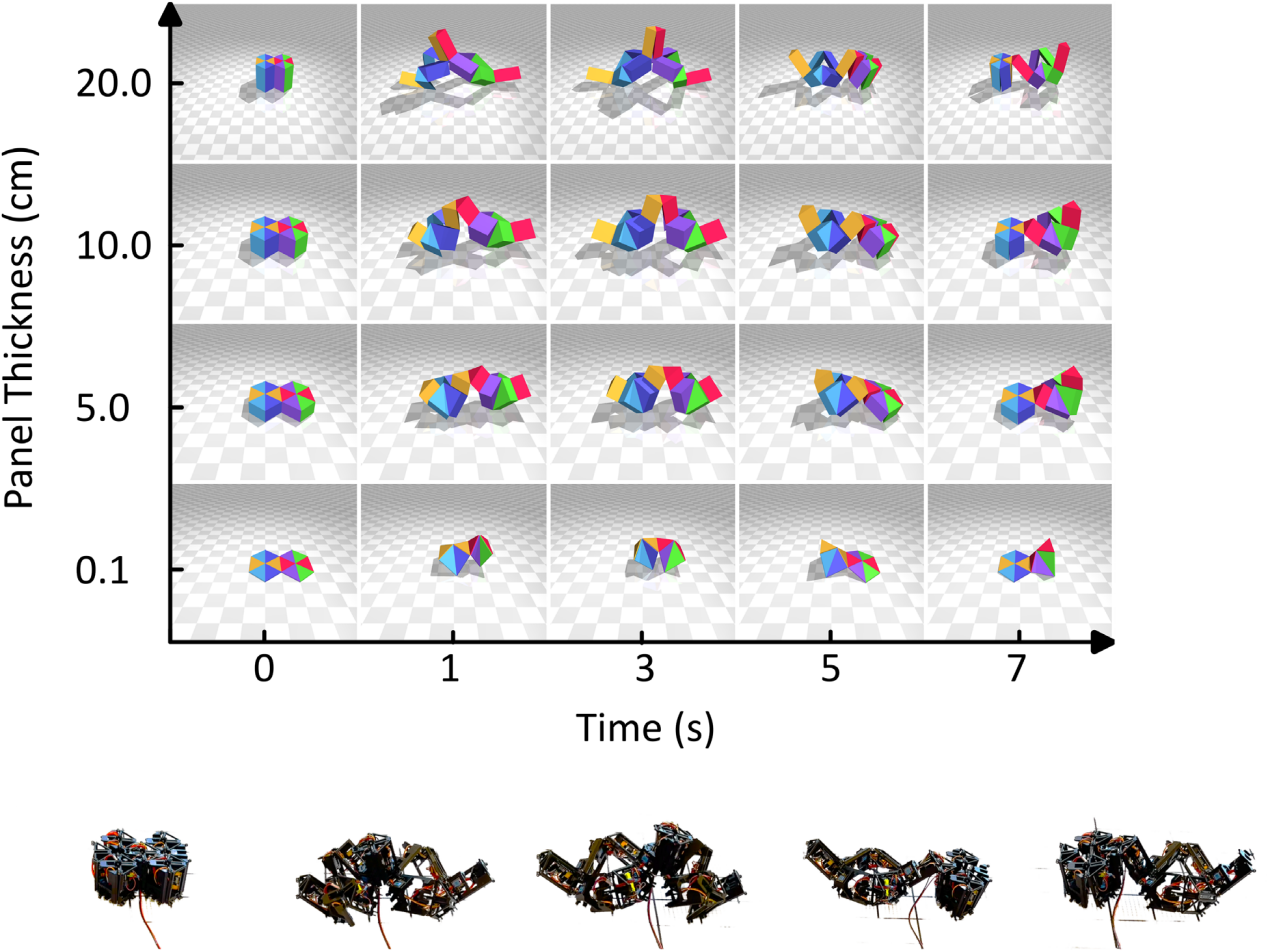
Our selected pattern based on a modified Yoshimura tessellation. The top four rows show the same pattern undergoing the motion of one cycle of a gait at different thicknesses. The row beneath the graph shows the physical robot undergoing the same gait. The robot has a panel thickness of 9 cm.

The robot is actuated using 13 servo motors and powered with onboard batteries. Control computations are performed by an onboard microcontroller (ESP32). The flat state of the robot serves as a kinematic branching point from which state the robot can enter 16 different kinematic branches, 7 of which are unique under rotational and mirror symmetry. This means that the robot changes its kinematic structure by passing through the flat branching point, and choosing a new kinematic branch. In each of these branches, the robot operates with three controllable degrees of freedom. We have developed an algorithm that automatically generates different gaits determined by different closed paths through a three‐dimensional action space. See Figure 2 for a visualization of three of these kinematic branches.

Our contributions are the following:

- We show that a thickness‐invariant origami architecture can internalize power, actuation, and control while preserving foldability for untethered operation.
- We establish the branch structure of the two‐cell mechanism and identify the symmetric reduction from 16 branches to 7 unique modes.
- We demonstrate that distinct branches correspond to distinct locomotion behaviors in both simulation and physical experiments.
- We provide a control and gait‐search framework for exploiting branch‐dependent motion in multi‐DOF origami robots.

The key novelty in this work is using the thickness‐invariant property of the Yoshimura pattern to construct a robot capable of locomotion that contains all requisite actuators and control components. We also include the explicit enumeration of the distinct kinematic branches that this robot can access, using the flat configuration as a change point. We show gait discovery and hardware validation for motion using unique branches, allowing the same thick‐origami robot to move in four different ways.

## 2 Related Work

Origami‐based robots have the potential to be incredibly versatile, as they can move and transform in ways that are uncommon in other robotics domains. One common challenge facing engineers attempting to adapt origami to their use cases is thickness accommodation: origami patterns traditionally come from media of negligible thickness, and engineering applications frequently require media of non‐negligible thickness.

### 2.1 Origami Robotic Systems

Understanding and applying the kinematics of origami mechanisms has enabled the development of robots capable of a range of unique movements within a range of interesting domains, including walking, swimming, and jumping [4, 5, 6]. Some origami patterns have used shape memory alloys and temperature‐responsive actuators to provide folding actuation in a low‐profile medium [7, 8, 9, 10]. A variety of materials are reviewed for use in active origami, including hydrogels, shape memory polymers, liquid crystal elastomers, and magnetically responsive materials [11]. Many of these robots use external actuation, including pneumatic actuation with an external pressure source [10, 12, 13] or external magnetic fields [14, 15]. Different origami‐based robots also explore different methods of integrating origami into robotics design, with some using a thin substrate to house origami‐based mechanisms that drive motion [16].

### 2.2 Thickness Accommodation in Origami

A wide variety of techniques have been developed to adapt origami patterns designed from thin sheets to work for thick materials [17]. Previous work with thickness‐invariant origami includes explorations into replacing hinges with specialized joints and adapting manufacturing processes, such as additive manufacturing, for constructing thickened models [18, 19]. Other work has explored various different patterns, which accommodate their own thickness purely through pattern geometry, and how these patterns could be used in different engineering applications [2, 3, 20]. These thickened patterns have also been utilized as deployable load‐bearing structures, with the Yoshimiura pattern used in [21], and a Miuri–Ori pattern with a series of cuts [22].

For thickened origami models, other work has focused on modeling the kinematics of open origami chains, computationally determining the optimal paths of movement for different reconfiguration scenarios [23]. Researchers have also explored the capabilities of folded patterns constructed from rolling‐contact joints and seen promising results for self‐contained origami robots [24].

Recent work has focused on using kinematic bifurcations for a gripper with multiple paths for the fingers [25], as well as for a millipede‐style robot [26]. Our work uses similar bifurcations but also uses the thickness of the panels to house mechatronic components, as well as optimizes for locomotion gaits along different paths of the bifurcations.

## 3 Methods

### 3.1 Kinematics

We developed a modular architecture where every panel of the robot is a triangular prism. This architecture allows the robot to move freely with all power storage and motion generation contained within the individual panels and allows for greater flexibility in distributing components throughout the robot. Controlling the robotic motion requires us to describe the kinematics of three things: how individual motors actuate a hinge from within the panels, how combinations of hinges can actuate a vertex within the pattern, and how different hinges can work together to move the pattern as a whole.

#### 3.1.1 Hinge Geometry and Kinematics

**FIGURE 3:**
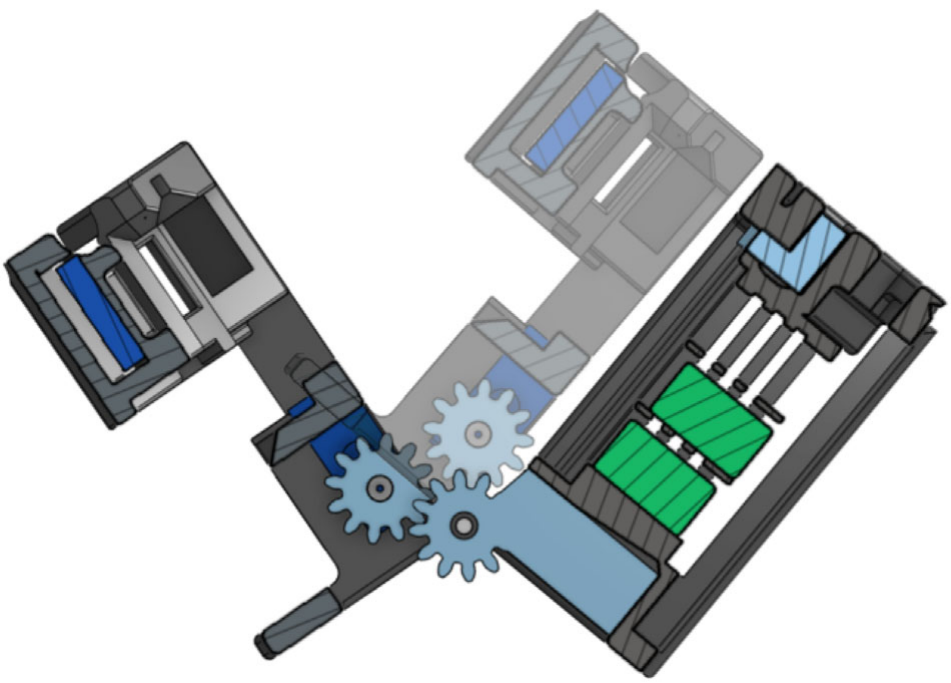
The gear assembly that actuates the hinges. For simplicity, and to match the 180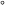 of rotation provided by the servo motors, the gears have a 1:1 ratio. A slight interference fit between the 3D printed gears ensures minimal backlash.

Our thick origami robot requires an actuation system that allows the panels to rotate about hinges at their corners, but it uses actuators that remain inside the volumes of the panels. Each hinge between panels of the robot is actuated by one servo motor embedded within the adjacent panel. The torque output of the servo is then translated to the hinge by two gears, each with a pitch diameter equal to the distance from the servo output to the hinge, meaning that the gears have a 1:1 ratio. Because the gear ratio between the servo output and the hinge is 1:1, we simply subtract the output values from this function from 180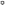 before sending them as control signals to the motors. As shown in Figure 3, one of the gears is attached to the servo, and the other only has three‐quarters of the teeth available, with the rest of the part extending into the adjacent panel.

#### 3.1.2 Vertex Geometry and Actuation

The next level of kinematic control is coordinating the motions of hinges that meet at a vertex of the pattern. Our panels are arranged in the undeflected position following the Yoshimura tessellation, where six fold lines of the flat pattern intersect at a point. In the case of the offset hinge technique used to accommodate panel thickness, the mountain folds are moved to the bottom of the panels, and the valley folds are located at the top [1]. The geometry of this pattern means that a Bennet linkage is embedded in the center of the pattern, allowing for 1 DoF motion around the vertex. To actuate one of the halves in this style, we fold along all six hinges concurrently. To do this, we use the following equation, modified from [1] to relate the angles of the four shorter hinges on the top of the pattern to the two longer hinges at the bottom of the pattern:

(1) $$\phi_{2}=2\cdot atan\left(\sqrt{2}\cdot tan\left(\frac{\phi_{1}}{2}\right)\right)$$

where $\phi_{2}$ is the dihedral angle around the shorter hinges, and $\phi_{1}$ is the dihedral angle around the longer hinges (see Figure 1). With this equation, we can coordinate the motion of all motors around one unit of the pattern to achieve the correct angles. This coordinated motion of the motors poses both a challenge and an opportunity. Folding all folds about a vertex requires all six motors to move in sync. Motors moving out of sync would make the motors fight each other, which would require wasted input energy and increased torque. However, if the motors are coordinated, they all contribute energy to the motion, meaning that loads are distributed among the motors, thereby minimizing torque.

#### 3.1.3 Pattern Motion and Kinematic Branches

Another opportunity presented by the thickness‐invariant pattern is the opportunity for kinematic branching, allowing the robot to switch between discrete kinematic configurations. From the flat state, any of the three pairs of hinges along a single fold line can fold together, or all of the hinges can fold together at once. Each of these four options is mutually exclusive: once the pattern has begun folding along one crease (or all of the creases at once), motion around the vertex reduces to a single degree of freedom.

**FIGURE 4:**
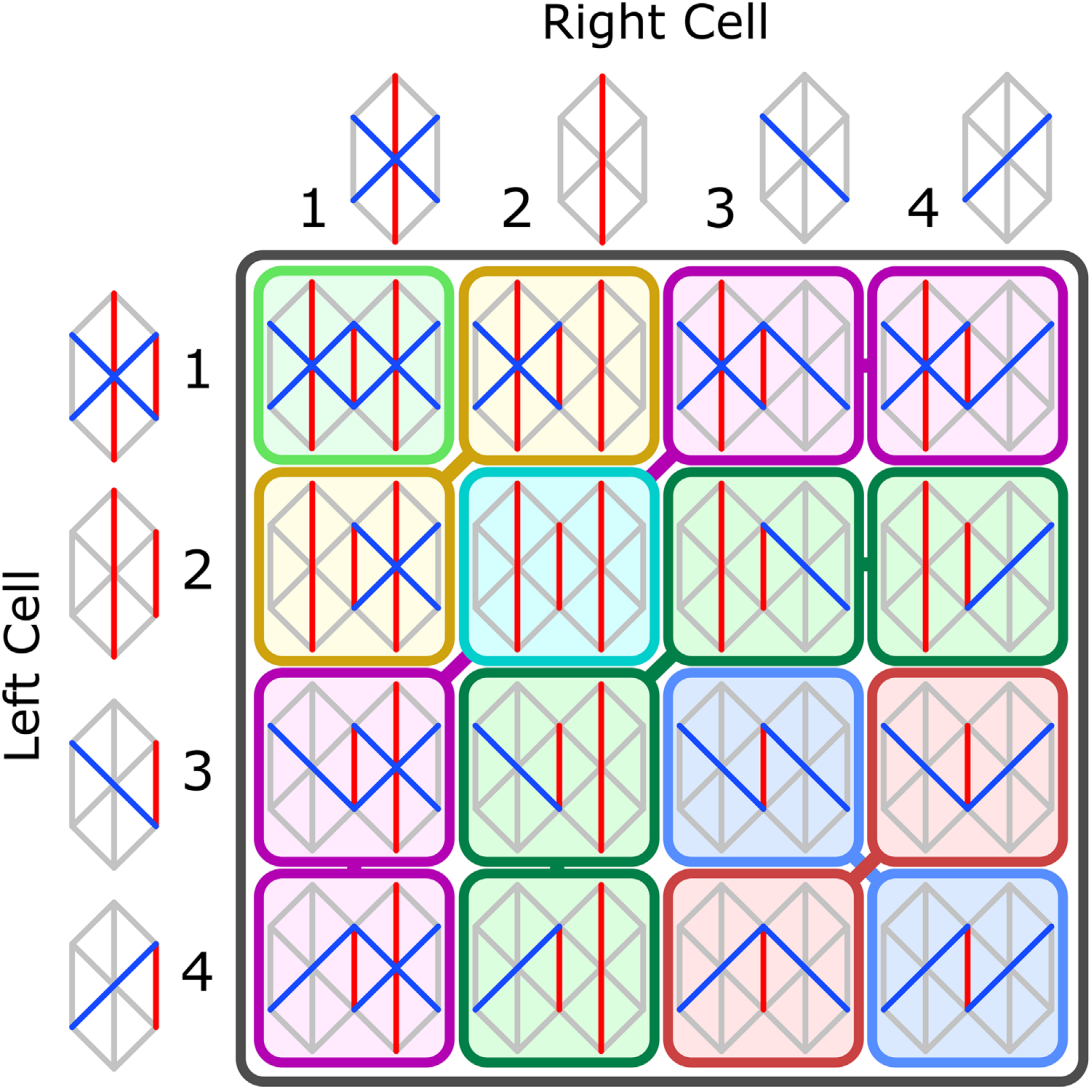
All 16 kinematic branches, with the 7 symmetric subgroups highlighted in different colors. Specific kinematic branches are named by their row and column, with the top right cell in the table being referred to as “(1, 4)”. The vertical axis encodes which kinematic branch is taken by the left vertex, while the horizontal axis encodes the branch taken by the right vertex. Note the 180
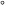
‐rotational symmetry of patterns on opposite sides of the diagonal, as well as the mirror symmetry of some patterns in the third and fourth rows and columns. Also note that the middle fold in all patterns is always present.

Because our chosen model contains two vertices of the Yoshimura pattern, with one hinge in the middle joining them, there are 16 total kinematic branches that the robot can access from the flat state. Due to rotational and mirror symmetry, these 16 branches can be reduced to 7 unique modes (see Figure 4). Each of these branches has three degrees of freedom, as each branch consists of a choice of fold for the left half, a choice of fold for the right half, and the middle fold, which is unaffected by the choices made on either side. These three degrees of freedom hold until collisions of the robot with itself occur, which depend on the specific choice of kinematic branch made.

### 3.2 System Design

**FIGURE 5:**
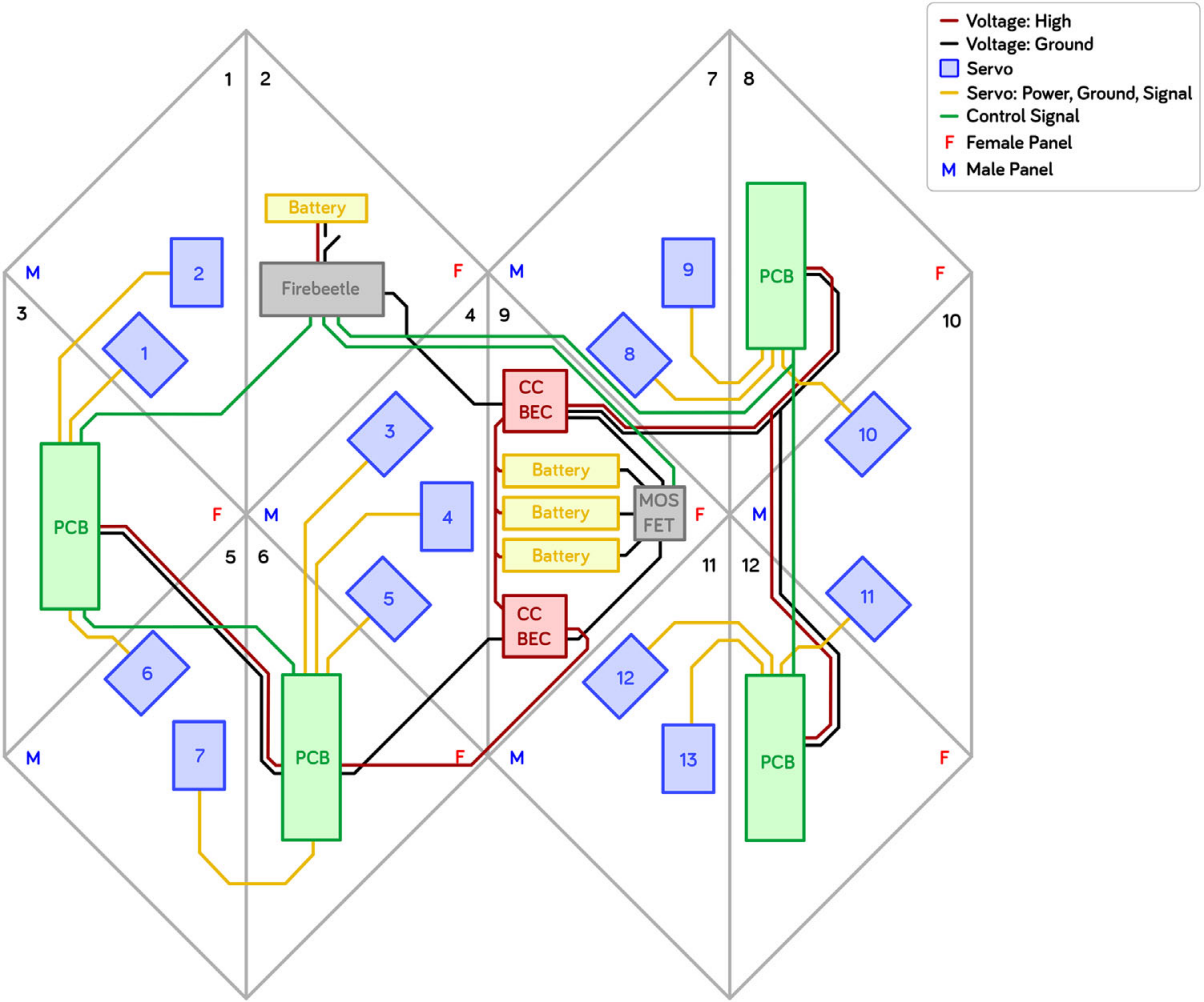
Component placement and wiring within the 12 panels of the robot. Signals sent from the microcontroller to the PCBs pass through, meaning the PCBs can be wired in series and obtain all necessary signals.

Our physical robot was designed to demonstrate the capabilities of the thickness‐invariant pattern. The primary design requirements were:

- Fully integrate all hardware components into the robot's structure (see Figure 5).
- Design for sufficient structural strength to withstand folding cycles.
- Ensure kinematics imitate the thickness‐invariant pattern.
- Produce controlled and repeatable movement profiles.
- Minimize overall system size.

These requirements guided the overall architecture of the system, informing both the structural layout and the selection of the actuation and control components. In particular, strict constraints were imposed by the internal hardware and geometric compatibility in space and linkage design. This was further compounded by the requirement for repeated motion stressing robustness and stiffness in the structure.

The system was therefore developed as a tightly integrated electromechanical design, where the mechanical structure, actuation strategy, and component placement were considered simultaneously rather than independently. The following sections describe how these requirements were addressed, beginning with the mechanical design of the structure.

#### 3.2.1 Mechanical Design

The mechanism is comprised of 12 triangular prisms that are connected via hinges to form the complete folding pattern. Each of these panels is based on a right triangle with edges lengths of 7.0, 7.0, and 9.9 cm, and has an extruded thickness of 9.0 cm. The panels are divided into two functional subgroups: male and female. All panels were designed to share identical external dimensions to simplify manufacturing and assembly. The male panels became the limiting case for the overall geometry, as they must house up to three servos, which are required to actuate all interfacing hinge connections. These servos interface with the corresponding female panels with their attached gears and gear arms to drive system motion, as described in the kinematics section. Because the male panels are so densely packed with servos, the female panels were used to store the necessary electrical components for the system.

#### 3.2.2 Hinge Design

Hinge joints were used to link the panels and facilitate the folding patterns. The hinge joints are formed using a metal rod with an interference fit on both ends of the joint and a clearance fit in the middle. This strategy fixes the rod in place while allowing the hinge to freely move with minimal friction, which adds up quickly in a design with dozens of hinges.

We designed the joints of the prototype for additive manufacturing by separately 3D printing hinges, which we bonded to the module with adhesive. By manufacturing the hinges separately, we minimized the number of failed 3D prints, ensured that interfacing components had ideal printing orientations, and saved both material and time for developing our final prototype.

The structural components were fabricated from polylactic acid (PLA) using a fused deposition modeling (FDM) 3D printer. Additive manufacturing was selected due to the geometric complexity of the design, particularly the need to integrate internal cavities, hinge features, and actuator mounting within a compact volume. PLA was chosen for its low cost, low density, and ease of fabrication, while still providing sufficient strength for the intended loading conditions.

The gears used for interfacing with the servo outputs were similarly printed in PLA and were secured to both the panels and the servos with press fits. The gear train consists of two parts with the same pitch diameter: one standard gear, fitted to the servo, and an arm with an integral sector gear that is fitted into the adjacent, female panel. This part, which has an interference fit of its own with its adjoining panel, ensures that the rotational output from the servo on the male panel is converted into rotation about the hinge axis without any backlash.

#### 3.2.3 Electrical Design

The electrical system provides power and control signals for the mechanism, facilitating motion while remaining fully contained within the internal volume of the structure. See Figure 5 for reference on how electrical components were connected to one another.

##### 3.2.3.1 Actuation Hardware

The mechanism is actuated by 13 servos: 12 metal‐geared MG90S's and a single Futaba S3170G. The servos, except the Futaba S3170G, drive the fold lines in pairs, as servos placed around vertices of the pattern are coupled to one another. This allows for the smaller, weaker servos to support one another during actuation. It also means that in any of the movement modes where all servos around a vertex are actuated, such as mode (1, 1), all six servos around that vertex work to support the load encountered during motion (see Figure 4 for gait description).

Servo selection was driven by the competing requirements of torque capacity, space constraints, and power consumption. The MG90S servos were selected for most hinge locations due to their compact size and relatively low current draw, while still providing sufficient torque when paired. At these joints, load sharing between two servos enables effective actuation without requiring larger actuators. In contrast, the central hinge interface (between panels 4 and 9, see Figure 5) experiences a significantly higher moment and is driven by a single actuator, necessitating the use of the higher‐torque Futaba S3170G servo.

This mixed‐servo approach provides the required torque across the mechanism while minimizing overall volume and power consumption. However, it introduces slight differences in dynamic response, as variations in internal control electronics and encoder resolution between servo models result in non‐uniform responses to identical input signals.

##### 3.2.3.2 Microcontroller Selection

The microcontroller for this system must be capable of directly controlling multiple servos, assisting rapid iteration during development, working with minimal power consumption, and inhabiting minimal space. In particular, the ability to generate independent control signals for all 13 servos without additional hardware was a key requirement, in order to conserve space and maintain simplicity for debugging. The FireBeetle 2 ESP32 was selected as it provides a sufficient number of GPIO pins to directly drive all servos, integrated WiFi for wireless code uploads, and efficient power usage in a small footprint. Alternative architectures were considered, including the use of dedicated servo driver boards. While these can simplify signal generation and improve scalability, they would have introduced additional hardware complexity, increased system volume, and adding weight. For future scaling of the same pattern/robot with more panels, secondary controllers may be considered advantageous. However, for this application, direct control from a single microcontroller provided a simpler and more efficient solution.

##### 3.2.3.3 Power Supply

While the control architecture met functional requirements, current limits and supply emerged as a critical design limitation during system development. Earlier prototypes experienced brown‐outs due to high current demand induced by simultaneous servo actuation. To mitigate this, two separate power systems were implemented with a shared ground reference.

The first power system was dedicated solely to the FireBeetle microcontroller to ensure constant power regardless of brownouts caused by the servos. Due to the controller's relatively low current requirements, a compact, low‐mass battery was sufficient. A 4.8 V, 200 mAh battery was selected to power the microcontroller to minimize added weight and volume.

The second power system was dedicated to the servos. This system required the ability to deliver high current on demand while maintaining a compact, mass‐efficient form factor. To direct battery selection, candidate options were evaluated based on their ability to fit within the panel geometry while providing high current output rates. Additionally, the battery voltage needed to meet or exceed 4.8 V to ensure proper servo powering.

For the second power system, we used three 7.4 V, 250 mAh LiPo batteries in parallel to power the system. The selected batteries were connected to two CC BEC 2.0 voltage regulators, which stepped the voltage down to a stable 6.0 V for the servos. This configuration provided sufficient current capacity while maintaining voltage stability under dynamic loading conditions.

The original intention of this design was to have batteries serve the electrical needs of the robot, but batteries with a capacity convenient for testing were unavailable at the time of writing. While batteries were able to power the robot, they could only do so for approximately 10 min, and so for the testing discussed in this paper, a tether to a power supply was used.

##### 3.2.3.4 Power and Signal Distribution

To distribute power and control signals throughout the mechanism, wiring was routed to custom PCBs that organize power and signals to the servos. This approach allowed compact power and signal lines to be routed to each board, rather than running individual wires directly to each servo, thereby reducing wiring complexity and minimizing constraints on motion. The PCBs then distribute power and signals locally to dedicated pin headers, enabling the servos to plug in directly. A total of four identical PCBs are integrated into the mechanism to standardize and simplify the signal distribution framework.

#### 3.2.4 Mechanical–Electrical Analysis

Early iterations of the mechanism measured high current draw from the servos during folding motion. To ensure adequate current supply to the system, a first‐order analysis was performed to relate mechanical loading to electrical demand.

The torque requirements at each hinge were estimated from the robot's approximate weight and linkage geometry using a static moment balance (τ=r·mg). Servo current draw was then estimated using manufacturer specifications, assuming a linear relationship between torque and current. The current at each hinge was determined as a fraction of the servo's rated maximum based on the ratio of required torque to maximum torque. From these torque values, the corresponding maximum current draw was calculated to size the required battery supply. These results indicate that the system operates well within both mechanical and electrical limits.

### 3.3 Control Strategies

We now present control strategies that explore the configuration space of the design and allow for simple forms of locomotion and reorientation. A key feature of our selected pattern is its ability to fold along several different kinematic branches. This allows the robot to take advantage of different modes for different movement needs. Instead of the robot being reconfigured externally, it can reorient itself to a different branch of the kinematic tree, enabling a different type of location. We present a framework for constructing gaits for different movement applications. This framework involves physics simulation, a gait parameterization for periodic gaits based on Bezier curves, and a genetic algorithm for gait discovery.

#### 3.3.1 Dynamics Simulation

We used a Python implementation of the MuJoCo simulator environment in order to efficiently find representative motions of the robot. We constructed an XML model of our robot, shown in Figure 2, keeping the dimensions of individual panels identical to the physical model, but without modeling any internal features. The MuJoCo model places hinge joints with actuators in between panels in a branching tree away from that panel. Kinematic closure of the loops around the two vertices is achieved by adding two equality constraints from one panel on the end of one branch of the tree to the panel directly opposite it, effectively forming a coincident constraint on the edges of those two panels. The locations that held the equality constraints held no actuators, as Mujoco actuators cannot be placed in locations without joints. Coordination for actuators on different joints of the same fold was achieved through control tendons, which ensured that forces applied to one fold were shared equally between the two actuators placed within it. For folds that consisted of one joint and two equality constraints, only one actuator was present, and it had the same strength as the actuators placed on all other joints.

The MuJoCo simulation was built to output both the final position and the cumulative orientation change of the robot across 64 s of simulation (32 000 time steps, at 2 ms per step), giving each gait policy time to move the robot away from its starting position and orientation. This number of time steps allowed for each gait to repeat eight times before the simulation ended.

Care was taken to ensure that the exact dimensions of the simulated robot matched those of the physical one, as well as the coefficient of sliding friction with the ground, the density of the panels, and the maximum torques provided by the simulated servo motors. As an incorrect value for the coefficient of sliding friction would result in highly unfounded simulation results, the sliding friction coefficient of the testing apparatus used was measured to be 0.3. This measurement was done with a weight, a sample 3D‐printed component matching panel geometry, and a spring scale.

#### 3.3.2 Gait Parameterization

**FIGURE 6:**
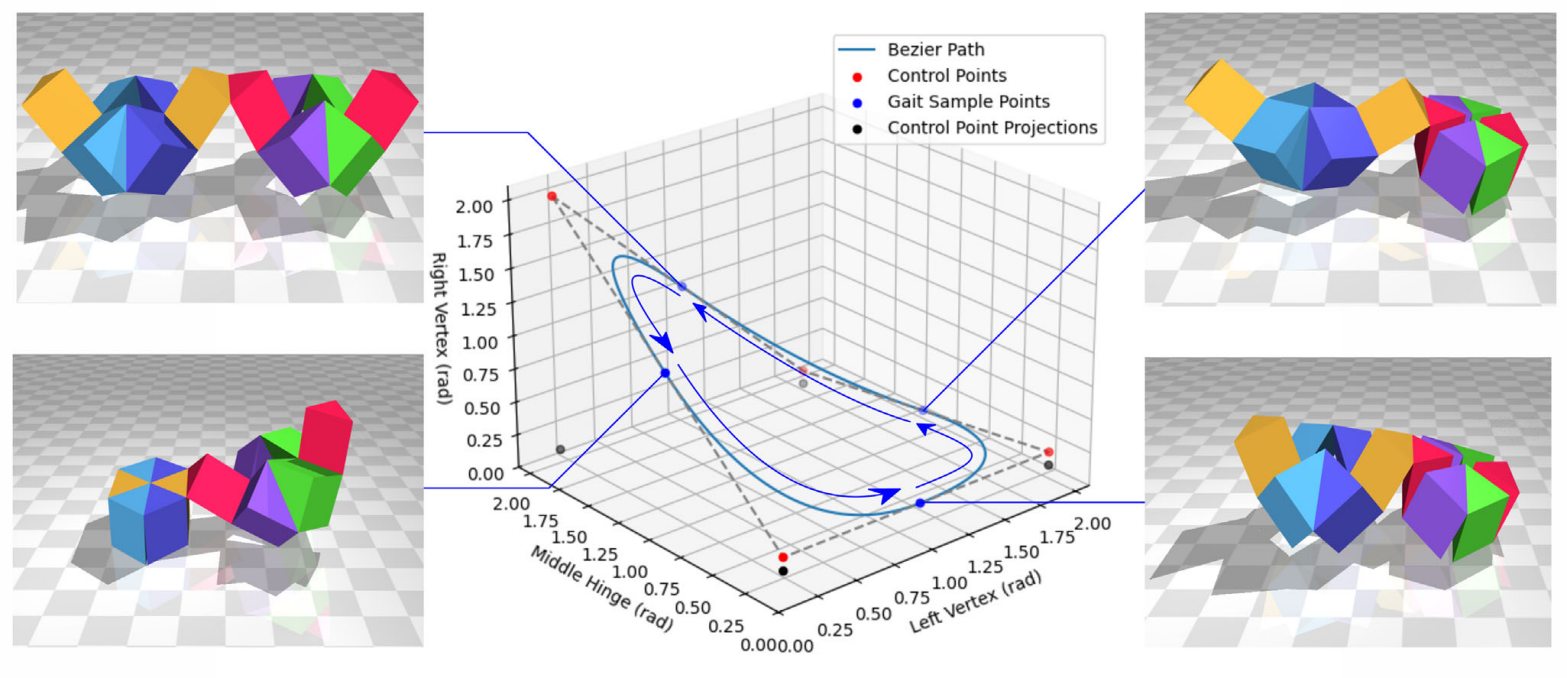
A visual representation of the path the hinges of the robot take through a three‐dimensional action space. This depiction represents the configuration used on the physical robot, which utilizes mode (1, 1) from Figure 4.

Regardless of the kinematic branch chosen for the robot to operate within, the robot always has three degrees of freedom in the angles of its hinges. This is the result of our choice of pattern: alternative variations on the Yoshimura pattern, as well as many other origami patterns, will likely have different numbers of degrees of freedom along different kinematic branches. In our case, this constant across all branches means that gait parameterization can be thought of as an exploration for periodic paths within 16 different three‐dimensional spaces. To find effective gaits, we developed a description for a closed Bezier curve in 3D space, which consisted of four control points, four interpolated tangent points, and four quadratic Bezier curves (see Figure 6).

#### 3.3.3 Genetic Algorithm

As there are seven symmetrically unique kinematic branches to explore, and there are three dimensions in the action space of each one, we used a genetic algorithm to optimize for different forms of motion. This choice was primarily due to the lack of a well‐defined gradient for our loss function, as well as the mix of discrete and continuous variables within well‐defined bounds. We used the Nevergrad optimizer [27], which provided simple but effective parameters for defining a search space and controlling the amount of CPU time used in exploring it.

**FIGURE 7:**
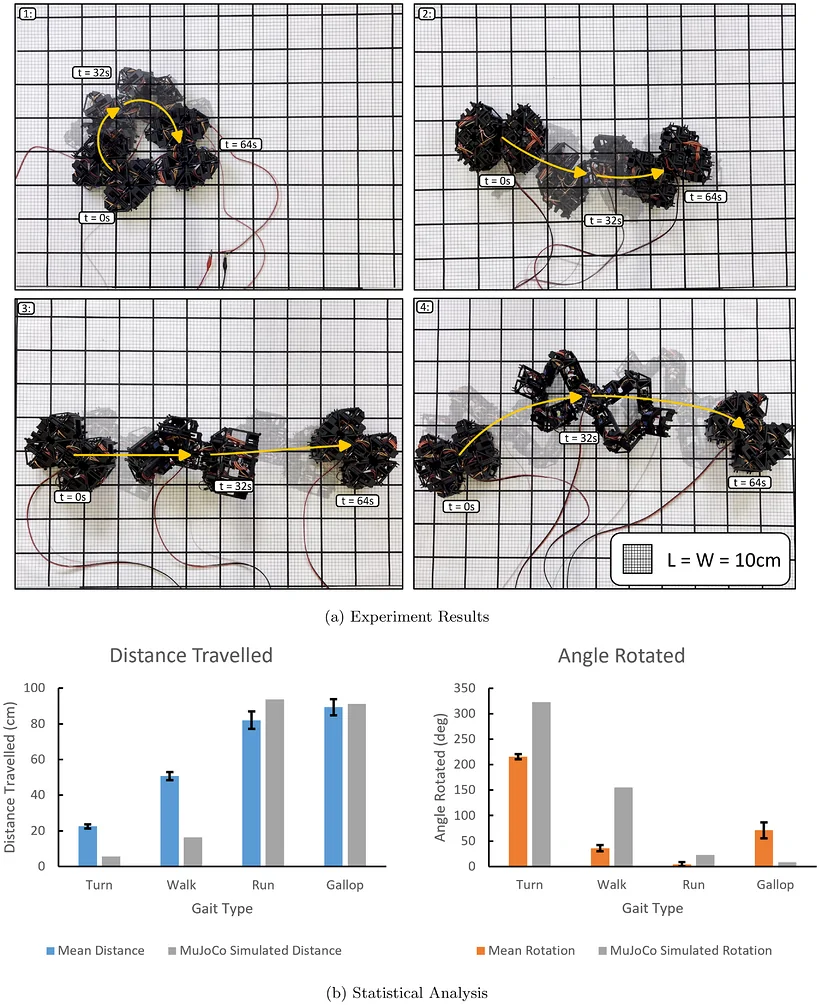
(a) The robot moving through four different gaits. Each image represents eight repetitions of a gait, with images taken every 16 s. The motion of the robot across each gait is highlighted in yellow. Gait 1: ”Turn” uses mode (4, 3) to turn in a tight circle. Gait 2: ”Walk” uses mode (3, 3) to crawl forward at a slow pace. Gait 3: ”Run” uses mode (2, 4) using one half the pattern to walk forward, and the other half as a counterbalance to shift the center of mass forward and backward across the gait. Gait 4: ”Gallop” uses mode (1, 1) and extends the body of the robot out the farthest. (b) The resultant statistical analysis of the experimental results. Measured values are in color, with error bars representing the standard deviation across seven samples. Values provided by the MuJoCo simulation of the gait are in gray.

Our gait exploration consisted of 42 individual optimizations, taking each of the 7 unique modes, placing it in the simulator both right‐side up and upside‐down, and optimizing for movement along the *x*‐axis, movement along the *y*‐axis, and rotation about the *z*‐axis. This optimization took four hours on an Intel 13900K CPU, with a budget of 2000 for each of the 42 sub‐optimizations. Once the gait exploration was completed, gaits were organized by their success in maximizing total distance traveled and total rotation achieved, and then the top four gaits with distinct locomotion modes were selected for experimentation. These gaits and their performance are shown in Figure 7.

## 4 Results

### 4.1 Locomotion

The unique structure of the robot allows it to achieve a variety of different shapes. Some shapes, such as mode (1, 1), allow for fast movement along the *y*‐axis, while others, such as mode (2, 4), allow for tight rotation around the *z*‐axis. This flexibility in locomotion means that the folding pattern can be programmed for different use cases, and can swap between different movement profiles using different kinematic branches available from the flat state. This implies that robotics designs based in more complex origami patterns will be capable of even more expression in motion, as they could easily have many more different folding modes.

As there are seven unique modes that the robot can operate in, two orientations (one right‐side up and the other flipped), and two loss functions (translation and rotation), we explored 42 unique optimizations to find which of the combinations were most successful at maximizing output movement over repetitions of the generated gaits. The results of our simulated exploration are in the Supporting Information. For experimentation, four gaits were chosen from this group of results for their mobility and distinctiveness in locomotion. These four gaits are referred to as Gait 1: “Turn” (4,3), Gait 2: “Walk” (3,3), Gait 3: “Run” (2,4), and Gait 4: “Gallop” (1,1).

Before each of these four gaits was run on the robot, they were experimentally verified in the MuJoCo simulation. The results of this simulation, and thus the expectation for the experiment, are summarized in Table 1.

**TABLE 1:** Experimental and simulated locomotion results.

	Distance		Rotation	
Gait	Experiment (cm)	Simulation (cm)	Experiment ( 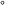 )	Simulation ( 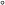 )
Turn (4,3)	22.50 (1.10)	5.62	215.43 (5.03)	323.16
Walk (3,3)	50.71 (2.26)	16.43	35.86 (6.14)	155.59
Run (2,4)	82.00 (4.88)	93.63	4.28 (4.07)	22.88
Gallop (1,1)	89.30 (4.54)	91.06	71.00 (16.00)	8.11

*Note*: Experimental values are reported as mean (standard deviation).

### 4.2 Physical Robot

For each of the selected gaits, the robot was run for 64 s, completing eight repetitions of each gait. This was repeated seven times per gait, to obtain a description of the behavior of each gait on the physical robot. The gait policies used on the robot were generated via simulation‐based optimization. This optimization provided repeatable, qualitatively distinct motions on the physical robot, but were not quantitatively predictive of the measured locomotion. The statistical results of this experimentation are shown in Figure 7 and in Table 1.

### 4.3 Repeatability Analysis

As shown in Figure 7 and in Table 1, the robot was able to move in a repeatable fashion. The simulation results generated by MuJoCo did not match the experimental results, especially toward the extremes of model locomotion. While some gaits showed close correspondence between simulation and experiment, others had substantial quantitative discrepancies, with differences reaching as much as a factor of seven in extreme cases.

**FIGURE 8:**
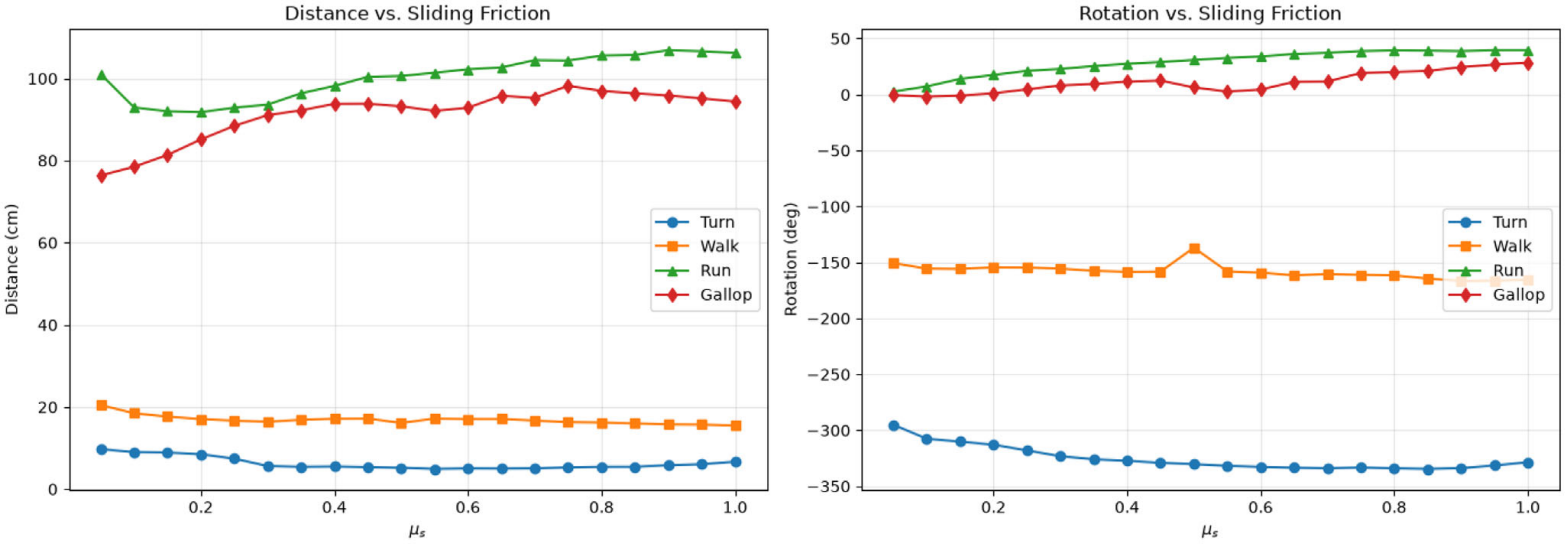
Functional relationships between simulated locomotion and ground friction. The coefficient of sliding friction was changed from 0.1 to 1.0 across 20 samples, and the resultant simulated locomotion was measured. While ground friction does affect the motion of the simulated models, their motion does not agree with the results found experimentally.

To better understand the sensitivity of the simulated model's locomotion to changes in the sliding friction coefficient, we ran a test across a range of values for $\mu_{s}$ and recorded the resultant values for distance traveled and total angle rotated, shown in Figure 8. Our results show a surprising insensitivity to the sliding friction coefficient, implying that the consistent disagreement between simulation and experiment is due to a sensitivity to another factor such as contact point geometry and asymmetry.

Based on observation of both the simulated motion and physical motion, it seems likely that some gaits are susceptible to slight changes in model geometry. Gait 4, the Gallop, exhibits bilateral symmetry at all points in time, yet in both the simulation and in experiment it demonstrated repeatable, non‐zero rotation to one side. This suggests a manufacturing asymmetry in the physical model, and a physics engine integration discrepancy in the simulation.

## 5 Discussion

Our work successfully demonstrates the feasibility of thickness‐invariant origami robots, and the ability to embed components into thick panels. We have demonstrated how movement policies can be generated and evaluated, and have shown how such a robot can be constructed and run. In so doing, we have shown how a thickness‐invariant pattern can be converted into a multi‐functional, mobile robot, with separate gaits in different modalities, which can be used to enable several unique forms of locomotion. We have also explored the limitations of generating movement policies in this fashion, and offered potential explanations as to the source of discrepancy between simulated and experimental results.

In this paper, we have focused on periodic gaits that exist only within a single kinematic branch. There may be useful motions that require moving the robot between different kinematic branches, meaning that the robot would pass through the flat state multiple times as it moves. This would also require reconfiguring the optimizer to optimize points within a set of different phase spaces, instead of only one, as well as choosing which subset of phase spaces, and in which order, the action state of the robot should pass through over the course of its gait.

Another way of making an origami‐based robot more expressive is by using more units of the underlying tessellation. Our robot contains two vertices of the Yoshimura pattern, linked by a single fold, but more vertices could have been added on either side, forming a longer chain of repeating units. This modular nature of tessellating origami patterns also lends itself well to the idea of self‐assembling units. Multiple simple units of a thickness‐invariant tessellation could combine into larger, more expressive robots at the external hinges. Alternatively, other thickness‐invariant patterns such as those described by Yang et al. [2, 3] could be used, resulting in various other modes of locomotion and interaction with the environment.

## 6 Conclusion

This paper shows how thickness‐invariant origami patterns can be applied to robotics, and explores the associated benefits. By choosing a simple pattern with several different kinematic branches, we were able to demonstrate how an origami‐based robot can reconfigure itself and move in multiple unique modes. The methods used to construct and control the robot are presented to provide an example of how we handled hurdles such as minimizing weight, cost, and footprint. We successfully demonstrated the capabilities of our robot by physically constructing and testing our design. The optimization and simulation algorithms worked well for identifying successful gaits and demonstrating their capabilities. Our results showed that the robot can locomote following the gaits generated through simulation, but requires better tuning of friction parameters to match the predicted speeds.

## Conflicts of Interest

The authors declare no conflicts of interest.

## Funding

This work was supported by internal funds from Brigham Young University.

## Data Availability

The data that support the findings of this study are openly available in GitHub at https://github.com/steel‐wing/origami‐robot.
